# Role of Forkhead Box O Transcription Factors in Oxidative Stress-Induced Chondrocyte Dysfunction: Possible Therapeutic Target for Osteoarthritis?

**DOI:** 10.3390/ijms19123794

**Published:** 2018-11-28

**Authors:** Rikang Wang, Shuai Zhang, Rahul Previn, Di Chen, Yi Jin, Guangqian Zhou

**Affiliations:** 1 Shenzhen Key Laboratory for Anti-ageing and Regenerative Medicine, Guangdong Key Laboratory for Genome Stability and Disease Prevention, Department of Medical Cell Biology and Genetics, Shenzhen University Health Science Center, Shenzhen 518060, China; wrk168ok@163.com (R.W.); zhangshuai2586@163.com (S.Z.); r.previn2903@yahoo.com (R.P.); 2 National Pharmaceutical Engineering Center for Solid Preparation in Chinese Herbal Medicine, Jiangxi University of Traditional Chinese Medicine, Nanchang 330006, China; 3 Department of Orthopedic Surgery, Rush University Medical Center, Chicago, IL 60612, USA; Di_Chen@rush.edu

**Keywords:** chondrocyte dysfunction, osteoarthritis, FoxO, oxidative stress, autophagy, aging, articular cartilage, molecular target

## Abstract

Chondrocyte dysfunction occurs during the development of osteoarthritis (OA), typically resulting from a deleterious increase in oxidative stress. Accordingly, strategies for arresting oxidative stress-induced chondrocyte dysfunction may lead to new potential therapeutic targets for OA treatment. Forkhead box O (FoxO) transcription factors have recently been shown to play a protective role in chondrocyte dysfunction through the regulation of inflammation, autophagy, aging, and oxidative stress. They also regulate growth, maturation, and matrix synthesis in chondrocytes. In this review, we discuss the recent progress made in the field of oxidative stress-induced chondrocyte dysfunction. We also discuss the protective role of FoxO transcription factors as potential molecular targets for the treatment of OA. Understanding the function of FoxO transcription factors in the OA pathology may provide new insights that will facilitate the development of next-generation therapies to prevent OA development and to slow OA progression.

## 1. Introduction

Osteoarthritis (OA), a leading cause of disability, is a prevalent rheumatic disease characterized by articular cartilage breakdown [1]. Chondrocytes are the major cell population in cartilage and they play an essential role in the homeostasis of cartilage metabolism. Oxidative stress, which disrupts cartilage homeostasis and thus contributes to the onset and progression of OA, occurs when the antioxidant capacity and autophagy level of chondrocytes are reduced. As a result, oxygen radicals are increasingly generated. Alternatively, chondrocyte oxidative stress can be induced when chondrocytes are exposed to an external source of reactive oxygen species (ROS) [2]. In osteoarthritic chondrocytes, increased ROS levels inhibit the phosphatidylinositol-4,5-bisphosphate 3-kinase (PI3K)/protein kinase B (AKT) pathway. Furthermore, it has been shown that ROS activate the mitogen-activated protein kinase (MAPK) pathway. The balance between the PI3K/AKT and MAPK signaling pathways is thought to play an important role in the initiation of the inflammatory process and the progression of OA [3]. Forkhead box Os (FoxOs) are a group of transcription factors downstream of both the MAPK signaling pathway and the PI3K/AKT pathway that regulate metabolic processes and OA progression [4].

The mammalian FoxO family consists of four main members: FoxO1, FoxO3, FoxO4, and FoxO6 [5], whereas human cartilage is only found to express FoxO1 and FoxO3 proteins [6]. As transcription factors, FoxOs regulate multiple gene expression and cellular functions, particularly those related to stress response, cell growth, cell survival, and longevity [7,8]. FoxOs are the main targets of the PI3K/Akt/serum- and glucocorticoid-inducible kinase (SGK) pathways activated by growth factor [9,10,11]. Akt may directly phosphorylate FoxO1, FoxO3, and FoxO4 at three conserved sites, resulting in nuclear export and the consequent functional inhibition [12]. FoxOs govern oxidative defenses such as manganese-dependent superoxide dismutase (MnSOD), catalase (CAT), and the DNA repair enzyme growth arrest and DNA damage 45 (GADD45) [13,14]. They can also regulate protein degradation mediated by the ubiquitin–proteasome system [15] and the autophagic/lysosomal pathway [16]. Changes in the expression and activation of FoxO have also been demonstrated in the pathogenesis of age-related diseases affecting bones [17], muscles [18], and the central nervous system (CNS) [19]. Recent studies have indicated the protective role of FoxO in oxidative stress-induced chondrocyte dysfunction and the pathogenesis of OA [6,20]. FoxO plays an important role in maintaining intracellular ROS homeostasis [21]. In chondrocytes, the dysregulation of FoxOs results in an increase in both the cell death rate and intracellular ROS levels [21]. Furthermore, interleukins (ILs) have been implicated in OA [22], and enhanced levels of interleukin-1β (IL-1β) in chondrocytes lead to phosphorylation and inactivation of FoxO1 [6]. FoxOs have also recently been shown to regulate cellular senescence signals [23], chondrocyte autophagy [20], chondrocyte maturation [24], and aging [6]. All of these physiological or pathological conditions contribute to the development of OA [25,26]. Therefore, it can be speculated that FoxO transcription factors are pivotal mediators of chondrocyte dysfunction. In this review, we will elucidate the link between OA and chondrocyte dysfunction, and we will focus on the impact of oxidative stress on chondrocyte maturation and dysfunction through FoxO regulation at the molecular level. We will also discuss alterations in FoxO expression and activation during OA development, the role of FoxOs in aging, and the inhibition of oxidative stress and inflammation in chondrocytes.

## 2. Role of Chondrocyte Dysfunction in OA

At the cellular level, chondrocyte dysfunction is the most apparent phenomenon in the pathogenesis of OA [27]. Chondrocyte senescence has been described as the major factor contributing to aging-related changes in cartilage homeostasis and function [28], and it has been associated with an increase in inflammatory mediators and matrix-degrading enzymes [29]. The selective elimination of senescent cells has recently been reported to attenuate the development of post-traumatic OA, reduce pain, and increase extracellular matrix (ECM) formation [30]. Chondrocytes are responsible for both the synthesis and the turnover of the ECM [29]. Senescent chondrocytes produce an abnormal ECM, typically characterized by increased stiffness [31]. These alterations of the ECM further promote OA pathogenesis by disrupting chondrocyte metabolism [31]. Chondrocyte autophagy is another homeostatic mechanism that acts through the removal of dysfunctional cellular organelles and macromolecules [25]. In experimental murine OA, the decreased expression of autophagy markers is correlated with the loss of ECM in cartilage, decreased autophagy in osteoarthritic chondrocytes, and an increase in apoptosis [28,32]. Interestingly, autophagy can be enhanced by rapamycin (a specific inhibitor of the mTOR) in human chondrocytes, leading to the arrest of OA development [33,34].

## 3. Oxidative Stress-Induced Chondrocyte Dysfunction

**Figure 1:**
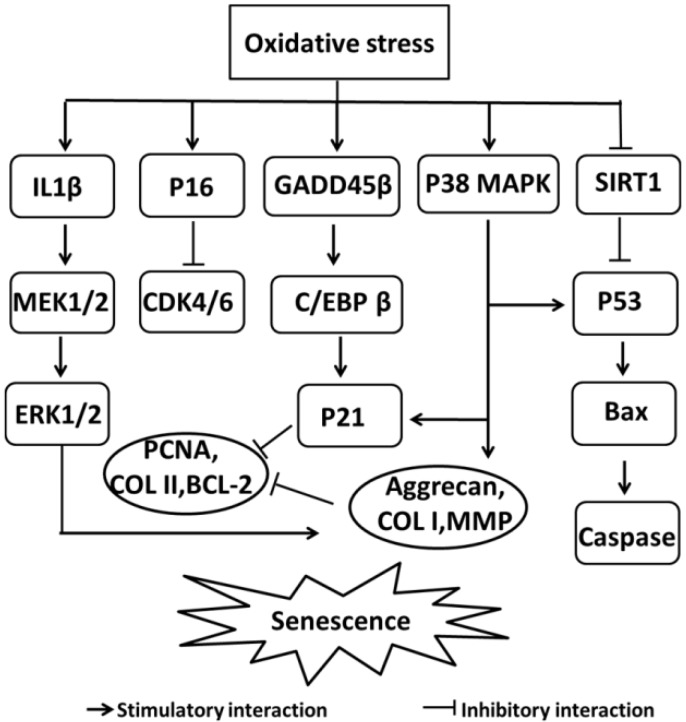
Signaling pathways of oxidative stress in chondrocyte senescence. Reactive oxygen species (ROS) are a main cause of senescence and they regulate signaling molecules, such as mitogen-activated protein kinases (MAPKs) (ERK/P38), P16, P21, and P53, which are eventually responsible for senescence. COL II = collagen type II, COL I = collagen type I, GADD45 β = DNA damage-inducible protein 45 β, C/EBP β = CCAAT/enhancer binding protein β, PCNA = proliferating cell nuclear antigen, MMPs = matrix metalloproteinases.

The most abundant ROS produced by chondrocytes are nitric oxide (NO) and superoxide anion (O^2−^), which themselves generate derivative radicals, including peroxynitrite (ONOO^−^) and hydrogen peroxide (H_2_O_2_) [35,36]. ROS are involved in modulating multiple signaling pathways, including those triggered by inflammatory cytokines and their receptors [37]. Increasingly, studies have shown that NO promotes inflammatory reactions by stimulating the production of proinflammatory factors and cyclooxygenase-2 (COX-2) in osteoarthritic tissues [38,39]. Osteoarthritic chondrocytes have a lower autophagic level and a higher ROS level compared to normal chondrocytes, and increased ROS levels have been found to inhibit autophagy in osteoarthritic chondrocytes [40]. By regulating the activation of the PI3K/AKT and c-Jun-N-terminal kinase (JNK) signaling pathways, ROS trigger the programmed cell death of chondrocytes [41]. NO also induces chondrocyte apoptosis by acting on both the inducible NO synthase (iNOS) and COX-2 systems, which are indirectly linked to the phosphorylation of mitogen-activated protein kinase kinase (MEK)1/2 and p38 [42]. Oxidative stress has been reported to induce cellular aging and accelerate the senescence of human chondrocytes [43]. As shown in Figure 1, ROS regulate several genes and signaling pathways that induce chondrocyte senescence [43], enhancing both the dedifferentiation and the senescence of chondrocytes through the extracellular-signal-regulated kinase (ERK) and p38 MAPK pathways [3]. IL-1β stimulates the expression of matrix metalloproteinases (MMPs) via activating ERK1/2 which in turn downregulates the type II collagen (COL II) and aggrecan expression in human chondrocytes [44]. P38 MAPK is also involved in matrix-associated proteins and COL II degradation in articular chondrocytes via MMPs and aggrecanases [45]. Moreover, oxidative stress induces chondrocyte senescence mainly by upregulating the expressions of p53 and p21 and by activating the p38 MAPK pathway [46]. P21 is involved in cellular senescence in the aging of articular cartilage through activation by GADD45 β and CCAAT/ enhancer binding protein β(C/EBP β) [47]. Moreover, proliferating cell nuclear antigen (PCNA) and COL II expression are negatively correlated with the p21 expression in cultured human articular chondrocytes [48]. Sirtuin 1 (SIRT1), a negative regulator of p53, prevents growth arrest, senescence, and apoptosis [49]. When oxidative stress increases, the upregulation of SIRT1 protects chondrocytes against DNA damage and telomere shortening [43]. P16 is another important factor associated with senescence. ROS are direct mediators of p16, and they promote senescence and dedifferentiation in OA cartilage and during in vitro terminal chondrogenesis [50]. Mainly, p16 engages in cell cycle arrest at the G1 stage by blocking cyclin-dependent kinase (CDK4)/6 [51].

**Figure 2:**
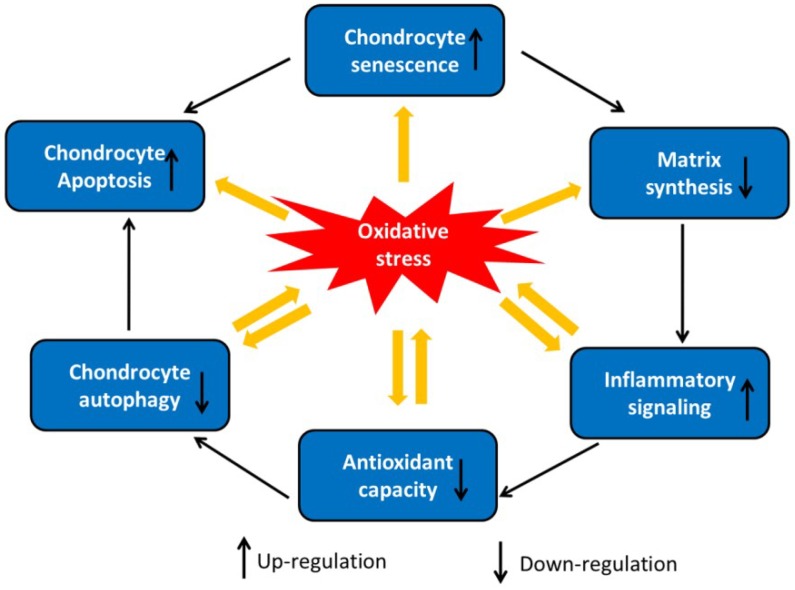
Inducers of oxidative stress in chondrocytes. Inducers of oxidative stress increase chondrocyte senescence, chondrocyte apoptosis, and inflammatory signaling and decrease chondrocyte autophagy, antioxidant capacity, and matrix synthesis.

Many studies have suggested that ROS inhibits the synthesis of new ECM in cartilage, leading to a loss of cartilage integrity [52]. COL II and proteoglycans are major components of cartilage ECM [53]. Within chondrocytes, ROS have been implicated in the inhibition of proteoglycans in both the superficial and deep zones by suppressing adenosine triphosphate (ATP) formation and mitochondrial oxidative phosphorylation [54,55]. ROS contributes to the loss of chondrocyte growth factor sensitivity and inhibits new ECM synthesis [56]. Oxidative stress inhibits the synthesis of chondrocyte proteoglycans induced by IGF-1 through the dual modulation of PI3K/AKT and MEK/ERK signal transduction [57]. Moreover, ROS inhibits the insulin receptor substrate-*1* (IRS-1)-PI3K/AKT signaling pathway and activates the ERK/MAPK pathway, which leads to a decrease in ECM synthesis and the suppression of the expression of aggrecan, COL II, and Sox9 [57]. ROS also reduce the protein expression of COL II by regulating the activation of the PI3K/AKT, p38, and JNK signaling pathways in rabbit articular chondrocytes [58]. When compared with normal tissue, the levels of p-JNK and p38 are higher in chondrocytes from human osteoarthritic cartilage. Furthermore, p38 MAPK is involved in the degradation of matrix-associated proteins and COL II in articular chondrocytes via MMPs and aggrecanases [45]. Figure 2 summarizes the role of oxidative stress in chondrocyte dysfunction.

## 4. Role of FoxOs in Oxidative Stress-Induced Chondrocyte Dysfunction

### 4.1. Regulation of FoxOs by Oxidative Stress

Overproduction of ROS promotes the nuclear localization of FoxO and its subsequent transcriptional activities [59]. In contrast, depending on the cellular context, oxidative stress enhances AKT activity, thereby inactivating FoxO as a result of the AKT-mediated phosphorylation of serine 256 in FoxO1 and serine 253 in FoxO3 [13,21]. The phosphorylation of FoxO by AKT or SGK involves the 14-3-3 protein, which functions as a scaffold within the cytoplasm. These kinases are thus sequestered within the cytosol, rendering them unable to bind to the specific binding sequence in gene promoters and to affect the transcription of target genes [60]. The expression of SGK, a negative regulator of FoxOs, is increased by p53 [61]. Following the stimulation with H_2_O_2_, JNK enhances the nuclear localization and transcription activities by the phosphorylation at sites (e.g., Thr447 and Thr451 in FoxO4) different from that by AKT [59]. Thus, it seems that distinct effects of AKT and JNK signaling play an important role in determining the functional activities of FoxO proteins [62].

**Figure 3:**
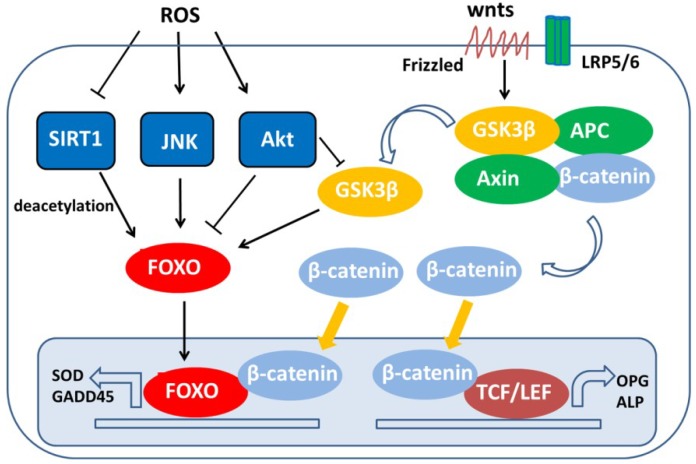
Activation or inhibition of Forkhead box O (FoxO) through ROS-induced posttranslational modifications. An increase in intracellular ROS induces the activation of the kinases JNK and Akt and inhibits the deacetylase SIRT1 and Wnt/β-catenin signaling. The dephosphorylation and acetylation status of FoxO can lead to the induction of a specific subset of genes that regulates cellular detoxification. FoxO = Forkhead box O transcription factor, ROS = reactive oxygen species, FRE = FoxO response element, SOD = superoxide dismutase, GADD45 = growth arrest and DNA damage 45, OPG = osteoprotegerin, TCF/LEF = T-cell-specific transcription factor/lymphoid enhancing factor, Axin = axis inhibition, APC = adenomatous polyposis coli.

AMP-activated protein kinase (AMPK) is an enzyme that monitors chondrocyte energy status and inhibits the pro-catabolic response of chondrocytes to biomechanical stress and inflammation. Importantly, AMPK signaling decreases with age [63]. AMPK exerts a chondroprotective effect, and studies have suggested that peroxisome proliferator-activated receptor gamma (PPARγ) coactivator 1α (PGC-1α) and FoxO3a are implicated in this process [64]. The levels of PGC-1α and FoxO3a are decreased in cartilage obtained from both aging mice and mice suffering from OA [64]. However, FoxO deacetylation occurs through SIRT1, which activates FoxO transcriptional activity. The decline of SIRT1 levels in response to small interfering FoxO (siFoxO) causes a decrease in FoxO transcriptional activity [65]. Consistently, resveratrol, an activator of SIRT1, inhibits inflammation and apoptosis and acts as an effective antioxidant in chondrocytes by upregulating FoxO transcriptional activity [66,67,68]. Hence, cells with decreased expressions of FoxO and SIRT1 proteins possess fewer antioxidant and autophagy proteins after exposure to oxidative stress. The main effector of the canonical Wnt signaling pathway is free β-catenin, which directly binds to T-cell factor (TCF) to form a transcription complex. Previous studies have suggested that cellular oxidative stress, as well as the overexpression of FoxO, leads to reduced binding between TCF and β-catenin, and it simultaneously increases the FoxO/β-catenin complex formation [69]. The binding of β-catenin with FoxOs forms a transcriptional complex that inhibits β-catenin to mediate transcription and to decrease osteoblastogenesis in vitro [70]. Additionally, the functional interaction between β-catenin and FoxO is evolutionarily conserved in oxidative stress signaling, and high ROS levels or growth factor depletion enhance the binding of FoxOs with β-catenin, which causes increased FoxO transcriptional activity in mammalian cells [71]. Smurf2-mediated proteasomal degradation of glycogen synthase kinase 3 beta (GSK-3β) results in the upregulation of β-catenin in chondrocytes and ultimately induces early events of OA in mice [72], and that inhibition of GSK3β might block chondrogenesis in vitro [73]. Furthermore, GSK3β activity is critical for the preservation of the chondrocytic phenotype and the maintenance of the cartilage ECM integrity, which is regulated by the classic Wnt signaling pathway. The short term of β-catenin upregulated in chondrocytes following to GSK3β inhibition may be adequate to induce osteoarthritis in vivo [74]. GSK3β is inhibited by AKT, so it was not surprising to see that GSK3 activates FoxO (Figure 3) [75]. However, the essential signaling pathways (FoxO, β-catenin, GSK3β, etc.) by which ROS contribute to the OA pathophysiology are complex and demand more investigations.

### 4.2. Role of FoxOs in Defending against Oxidative Stress

Oxidative stress occurs when the balance between antioxidant defenses and the production of ROS is altered, resulting in a disruption of redox signaling [36]. Free radicals are detoxified by ROS scavengers present in chondrocytes, and the detoxification of cells during oxidative stress is mediated by antioxidant enzymes and non-enzymatic molecules that specifically scavenge various kinds of ROS [76].

ROS production and oxidative stress have been found to be elevated in patients with OA. Furthermore, antioxidant enzymes, such as superoxide dismutases (SODs), CAT, and glutathione peroxidases (GPX), are decreased in OA patients [77,78], indicating that deficits in antioxidant defense related to low levels of antioxidants may contribute to cartilage aging and OA development. GADD45 protein levels are also markedly lower in osteoarthritic tissue. Thus, osteoarthritic cartilage shows more ROS-induced DNA damage when compared to normal cartilage [79]. The most significant role of FoxOs is in the cellular response to oxidative stress [80]. Increased ROS levels can enhance the expressions of FoxOs [21], and FoxOs upregulate several antioxidant enzymes, such as CAT, MnSOD, and GPX (Figure 3) [13,24]. The downregulation of FoxO in human chondrocytes results in increased intracellular oxidative stress and ROS-induced apoptosis, along with reduced levels of ROS scavengers, such as GPX-1 and CAT, and autophagy proteins, such as Beclin1 and LC3 [21]. On the other hand, FoxO transcription factors regulate the transcription of genes related to DNA repair, such as GADD45 (Figure 3) [68]. These data support the hypothesis that FoxOs play an important role in maintaining the intracellular ROS balance and in stress resistance (Table 1).

### 4.3. Expression Patterns of FoxO in Articular Cartilage under Normal and OA Conditions

The expression and activation of FoxO transcriptional factors are highly context- and cell lineage-specific [17,18]. FoxO1 and FoxO3 proteins are highly expressed in normal human and mouse cartilage, whereas FoxO4 expression is very weak in these tissues [82]. FoxO1 and FoxO3 are primarily localized in nuclei and are expressed most commonly in the superficial and mid-zones of articular cartilage [6]. MnSOD, one of the major FoxO target antioxidants, is abundantly expressed in the superficial zone of human cartilage [83]. In contrast, FoxO1 and FoxO3 expression have been reported to be markedly reduced in the superficial zone, and their increased phosphorylation has been observed in cluster-like chondrocytes aggregated in fibrillated lesions [6]. These data support the concept that abnormal expression and activation of FoxOs are involved in the pathogenesis of OA. Recently, it has been found that the FoxO role in maintaining postnatal articular cartilage integrity is mediated by activating cellular defense mechanisms and regulating the expression of proteoglycan 4 (PRG4), an essential protein in cartilage lubrication and superficial zone protection [81] (Table 1). This further supports the pathogenic significance of FoxO reduction in OA-affected cartilage and suggests that FoxO protects against OA onset and delays disease progression.

FoxO activity is negatively regulated by the insulin/IGF-1 pathway, which acts through the PI3K- and AKT-mediated phosphorylation of FoxO. Both IGF-1 and IGF-1 receptors are highly expressed in human osteoarthritic cartilage, with even a higher expression in chondrocytes [84,85]. When compared with chondrocytes of the middle and deeper zones, the level of IGF-1 receptors are lower in the superficial zone [86]. These observations strongly indicate that a similar pattern of FoxO distribution may exist.

### 4.4. Role of FoxOs in the Regulation of Inflammation in Chondrocytes

FoxO proteins have been reported to be involved in signal transduction pathways related to inflammation [87]. IL-1β, a proinflammatory cytokine, can reduce the expressions of FoxO proteins and increase their phosphorylation in cultured human chondrocytes (Table 2) [6]. The phosphorylation of FoxO1, FoxO3, and FoxO4 is increased in chondrocytes stimulated with bFGF, PDGF, and the oxidant *tert*-butyl hydroperoxide (t-BHP) (Table 2) [6]. These phenomena are similar to those observed in rheumatoid arthritis synovial tissue, such as synovial cells and macrophages, in which FoxO1 and FoxO4 are phosphorylated after stimulation with IL-1β and TNF-α [88]. Moreover, TNF-α stimulates chondrocyte apoptosis and upregulates the mRNA levels of apoptotic genes through FoxO1 activation [89,90]. Silencing FoxO1 using siRNA in vitro significantly reduces TNF-α-induced apoptosis and caspase activity in ATDC5 and C3H10T1/2 cells differentiated by BMP-2 (Table 2) [90].

Among the inflammatory genes expressed in chondrocytes, the mRNA levels of ADAMTS-4 (responsible for the catabolism of aggrecan) and chemerin genes were elevated in chondrocytes transfected with FoxO siRNA. IL-1β further enhanced the expressions of ADAMTS-4 and chemerin when the FoxO expression was knocked down [21] (Table 1). The promoters of ADAMTS-4 and chemerin do not have FoxO DNA-binding domains. However, it has been suggested that the direct association between FoxO proteins and other transcription factor families (such as CCAAT/enhancer binding protein, Smad3/4, and STAT-3) can either activate or repress the transcription of diverse downstream target genes, thus participating in various cellular functions independent of FoxO DNA binding [14]. These findings may explain how the knockdown of FoxO can affect the expressions of ADAMTS4 and chemerin in cells stimulated with IL-1β.

### 4.5. FoxOs Regulate the Proliferation, Maturation, and Matrix Production of Chondrocytes

During endochondral ossification, chondrocytes undergo a series of remarkable events including proliferation, maturation, hypertrophy, and eventual apoptosis [91]. In the absence of FoxO, growth plate chondrocytes show an increased hypertrophic zone length in neonates and three-week-old mice, a highly disorganized growth plate in eight-week-old animals, and skeletal deformation at older ages [24]. Strikingly, a similar phenotype was observed in mice with a chondrocyte-specific deletion of phosphatase and tensin homolog (PTEN) from chromosome 10, which acts as an upstream inhibitor of FoxOs by regulating the activation of AKT [92,93]. These results demonstrate that the PTEN/FoxO axis is crucial for normal endochondral ossification. When compared with resting and proliferating chondrocytes, pre-hypertrophic and hypertrophic chondrocytes display elevated ROS levels [94]. In contrast, two-week-old mice that underwent treatment with the ROS scavenger *N*-acetylcysteine (NAC) from birth, showed decreased ROS levels in the growth plate and a reduction in the length of the hypertrophic zone [94]. These data further support the notion that FoxOs are crucial regulators of the oxidative stress defense in chondrocytes.

The proliferation and maturation of chondrocytes and the production of the cartilage matrix largely depend on the dynamic balance among GSK3β, mTOR, and FoxOs, all of which are downstream signaling molecules of AKT. GSK3β inhibition leads to extracellular matrix remodeling, mitochondrial dysfunction, and the terminal differentiation of chondrocytes, suggesting that GSK3β activity is important for articular cartilage homeostasis [95]. The Akt/FoxO signaling pathway enhances proliferation, but inhibits the maturation and matrix production of chondrocytes, indicating that FoxOs promote chondrocyte maturation and inhibit chondrocyte proliferation [96]. Several downstream target genes of FoxO, such as p27, Bim-1, and FasL, play an important role in chondrocyte proliferation and apoptosis [82,97] (Table 1).

### 4.6. FoxOs Regulate Chondrocyte Autophagy

Because the induction of autophagy decreases intracellular ROS, it protects chondrocytes from ROS and inflammation-induced injury [98]. In contrast, the inhibition of autophagy results in an increase in intracellular ROS and in the rate of apoptosis [20]. Additionally, autophagy is involved in the pathological process of OA that is responsible for the generation of ROS and reactive nitrogen species (RNS) [99]. FoxO proteins are regulators of autophagy, working as transcriptional activators of several proteins involved in autophagy, such as LC3 and Beclin1 [100]. The knockdown of FoxO1 and FoxO3 resulted in a significant reduction in the levels of LC3 and Beclin1, which were increased by stimulation with the oxidant *t*BHP [21]. Moreover, chondrocytes transfected with FoxO siRNA displayed a significant increase in apoptosis accompanied by caspase activation [20]. In contrast, the active form of the FoxO3 protein increases cell viability and induces the transcription of Beclin1 and LC3 in response to oxidative stress, suggesting that FoxO proteins support oxidative stress resistance in part by regulating the production of autophagy proteins in human chondrocytes [21] (Table 1). Furthermore, FoxO1 downregulation suppresses SIRT-1, which regulates oxidative stress and the autophagy process by post-transcriptionally modifying FoxO and p53 [101]. In addition to FoxO, SIRT1 may also influence autophagy directly by promoting the deacetylation of autophagy-related 5(ATG5), 7, and 8, which are key components of the autophagy network [102].

Autophagy regulates the expressions of genes involved in OA through the modulation of apoptosis and ROS [98]. Dexamethasone (Dex) increases intracellular ROS levels, the expressions of autophagy markers, and FoxO3 [20]. The knockdown of FoxO3a by siRNA reduced Dex-induced autophagy and increased Dex-induced apoptosis in chondrocytes. Additionally, silencing FoxO3 also increased ROS levels because FoxO3 is associated with reduced levels of antioxidant proteins [20,21]. These observations suggest that autophagy protects chondrocytes from apoptosis induced by glucocorticoids through the activation of ROS/AKT/FoxO3 signaling. Decreased autophagy contributes to cell death during the gradual degradation of cartilage [25]. Thus, autophagic activity decreases with age and may be responsible for cytoprotective effects in young cartilage [103].

### 4.7. Role of FoxOs in Aging and Longevity

Oxidative stress limits lifespan. Accordingly, an increase in oxidative stress resistance in invertebrates correlated with an increase in their lifespan [104]. For example, life expectancy in *Caenorhabditis elegans* (*C. elegans*) is increased when the expression of MnSOD or CAT is enhanced [105]. Kaempferol is a flavonoid with antioxidant activity that may translocate Forkhead transcription factor/DAF-16 into the nucleus leading to an increase in the *C. elegans* lifespan [106]. Similarly, the attenuation or disruption of the insulin or IGF-1 signaling pathways in mice or rats leads to a prolonged lifespan [107]. Mice in which IGF-1 and insulin signaling are reduced live longer than normal littermates, and they exhibit a general decrease in the pathological changes associated with aging [108]. Further evidence indicates that any major change in the IGF/AKT/FoxO signaling pathway in chondrocytes of the osteoarthritic human knee, combined with reduced FoxO and downstream stress response genes, is accompanied by increased cell damage [6,21]. During aging, a decrease in the FoxO expression in the lumbar intervertebral disc (IVD), which precedes the major histopathological changes related to lumbar IVD degeneration, is accompanied by a decrease in the expressions of sestrin 3 and SOD2 [109].

The hallmarks of skeletal degradation are a reduction in bone formation and an increase in bone marrow adiposity as age increases. Decreased bone mass in the elderly is related to a decrease in osteoblasts and an increase in myelodysplastic syndromes. These changes are related to an increase in oxidative stress and a decrease in growth factors, which activate FoxO transcription factors [110]. Wnt/β-catenin/TCF signaling stimulates bone formation and the inhibition of adipogenesis [111]. In turn, FoxOs inhibit Wnt/β-catenin signaling by transferring β-catenin from TCF to FoxO [112]. Moreover, Wnt signaling can be stimulated by SIRT1-induced deacetylation of FoxOs. Thus, a decline in SIRT1 activity in osteoblast progenitors may contribute to the age-related loss of bone mass [113]. It has been reported that there is an evolutionarily conserved interaction of β-catenin with FoxO transcription factors, which are regulated by insulin signaling [71]. Therefore, a link between FoxO and Wnt/β-catenin signaling in age-related OA is possible and needs further investigation.

**Figure 4:**
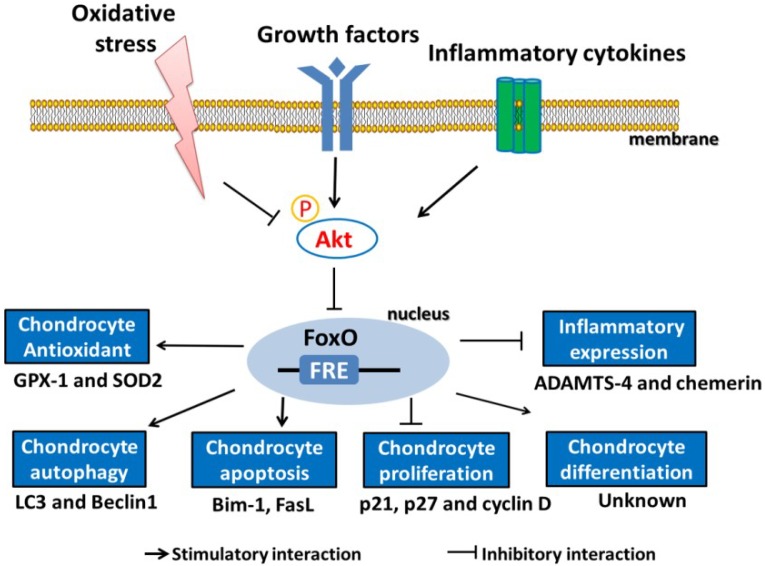
The protective role of FoxOs in osteoarthritic chondrocytes. In chondrocytes, FoxO activity is affected by three factors: growth factors (such as IGF-1 and insulin) and inflammatory cytokines (such as IL-1β and TNF-α) stimulate Akt activity, and the activation of Akt leads to the phosphorylation and inactivation of FoxO. During oxidative stress, the inhibition of Akt activity induces the nuclear accumulation and activation of FoxO. In the nucleus, FoxO recognizes and binds to FoxO response element (FRE). Activated FoxO thus regulates the transcriptional activity of its downstream targets, making possible the regulation of many chondrocyte processes, including proliferation, differentiation, and autophagy; apoptosis; and antioxidant capacity. FoxO = Forkhead box O transcription factor, IGF-1 = insulin-like growth factor-1, IL-1β = interleukin-1β, TNF-α = tumor necrosis factor-α, Akt = protein kinase B, FRE = FoxO response element.

It has been demonstrated that FoxOs regulate autophagy during the pathological processes of aging and OA [20]. Thus, FoxOs are probably ideal targets for therapeutic approaches that aim to modulate intracellular ROS levels. The roles of FoxO transcription factors in osteoarthritic chondrocytes are summarized in Figure 4. FoxO target genes are involved in the regulation of phenomena found in the pathology of OA, including chondrocyte apoptosis, proliferation, autophagy, and resistance to oxidative stress.

## 5. Conclusions and Future Perspectives

The progression of OA is closely associated with oxidative stress and ROS. The accumulation of intracellular ROS can disturb the anaerobic metabolism of chondrocytes and disrupt the homeostasis of cartilage [2,114]. Excessive levels of ROS harm the mitochondria and lead to further oxidative stress. Moreover, antioxidant defense mechanisms are weakened in OA [36,83,115], affecting chondrocyte phenotype [116], cell death [117], chondrosenescence [43,118,119], and aging [120,121] as well as the key mechanisms involved in both the initiation and progression of OA. Hence, the management of ROS levels in chondrocytes should be an effective strategy for the prevention and arrest of OA.

FoxOs are very important in the process of cartilage formation, and the lack of FoxOs leads to chondrocyte hypertrophy and abnormal cartilage [24]. However, the FoxO target genes that are involved in chondrocyte maturation are still unknown. Moreover, the possibility that FoxOs and their interacting proteins can act as potential therapeutic targets for OA requires further experimental validation. A recent study using RNA interference to screen for kinase and phosphatase regulators of dFoxO (Drosophila FoxO homolog) in Drosophila S2 cells identified GSK3β as one of the regulators of dFoxO [75]. A sequence search also revealed that GSK3 consensus phosphorylation sites (S/TXXXS/T) exist throughout the FoxO3a protein sequence, indicating that FoxO3a is a GSK3 substrate candidate [122].

Most studies concerning FoxOs are currently performed in cellular and animal models. Direct data from patients with OA are still needed and future work should study the expression of FoxOs in individuals with OA. The abnormal expression and activation of FoxOs in osteoarthritic cartilage have been reported to be involved in the pathogenesis of aging and OA [6]. Thus, studies of the genetic link between FoxOs and OA will provide evidence to support the role of FoxOs in this very important area of human pathophysiology. Furthermore, because multiple signaling pathways regulate the activity of FoxO transcription factors in response to oxidative stress in OA, it is necessary to elucidate how these diverse signaling pathways coordinate their effects to regulate FoxO activity.

Disease-modifying drugs for OA are rare and the usefulness of the currently available OA drugs is limited by the lack of adequate data on efficacy and safety. A better understanding of the underlying molecular mechanisms of OA promises to open new avenues for drug discovery. Some symptomatic slow-acting drugs on OA, such as Diacerhein and Rhein, modify the phosphorylation of FoxOs and reduce the deleterious effects of IL-1β on OA cartilage by inhibiting the expression of degrading enzymes [82,123]. Thus, FoxO transcription factors are critical regulators of the fate of chondrocytes and may have a protective effect during oxidative stress-induced chondrocyte dysfunction. Thus, targeting FoxOs and their signaling pathways may be an important therapeutic strategy for the treatment of OA.

## Figures and Tables

**Table 1:** The targets of FoxOs involved in the regulation of chondrogenesis and cartilage hemostasis.

Transcription Factors	Targets	Effects	References
FoxO1, FoxO3	CAT, MnSOD, GPX-1	Upregulation, antioxidant	[13,24]
FoxO3	GADD45	Upregulation, DNA repair	[68]
FoxO1	PRG4	Upregulation, cartilage homeostasis	[81]
FoxO1, FoxO3	ADAMTS-4, chemerin	Downregulation, inflammation	[21]
FoxO1, FoxO3	p21, p27, cyclin G2	Upregulation, proliferation	[82]
FoxO1	Bim-1, FasL	Upregulation, apoptosis	[82]
FoxO1, FoxO3	Beclin1, LC3	Upregulation, autophagy	[21]

**Table 2:** Effects of different protein factors on FoxOs in cultured human chondrocytes.

Protein Factors	Transcription Factors	Expression	Transcriptional Activity	References
IL-1β	FoxO1, FoxO3, FoxO4	Suppress	Downregulation	[6]
TNF-α	FoxO1	Suppress	Upregulation	[6,90]
TGF-β	FoxO1	Increase	No effect	[6]
PDGF	FoxO1, FoxO3, FoxO4	Increase (FoxO3)	Downregulation	[6]
bFGF	FoxO1, FoxO3	No effect	Downregulation	[6]

## References

[1] Taruc-Uy R.L., Lynch S.A. (2013). Diagnosis and treatment of osteoarthritis. Primary Care.

[2] Collins J.A., Wood S.T., Nelson K.J., Rowe M.A., Carlson C.S., Chubinskaya S., Poole L.B., Furdui C.M., Loeser R.F. (2016). Oxidative Stress Promotes Peroxiredoxin Hyperoxidation and Attenuates Pro-survival Signaling in Aging Chondrocytes. J. Biol. Chem..

[3] Yu S.M., Kim S.J. (2015). The thymoquinone-induced production of reactive oxygen species promotes dedifferentiation through the ERK pathway and inflammation through the p38 and PI3K pathways in rabbit articular chondrocytes. Int. J. Mol. Med..

[4] Ntoumou E., Tzetis M., Braoudaki M., Lambrou G., Poulou M., Malizos K., Stefanou N., Anastasopoulou L., Tsezou A. (2017). Serum microRNA array analysis identifies miR-140-3p, miR-33b-3p and miR-671-3p as potential osteoarthritis biomarkers involved in metabolic processes. Clin. Epigenet..

[5] Accili D., Arden K.C. (2004). FoxOs at the crossroads of cellular metabolism, differentiation, and transformation. Cell.

[6] Akasaki Y., Hasegawa A., Saito M., Asahara H., Iwamoto Y., Lotz M.K. (2014). Dysregulated FOXO transcription factors in articular cartilage in aging and osteoarthritis. Osteoarthr. Cartil..

[7] Liang B., Moussaif M., Kuan C.J., Gargus J.J., Sze J.Y. (2006). Serotonin targets the DAF-16/FOXO signaling pathway to modulate stress responses. Cell Metab..

[8] Tia N., Singh A.K., Pandey P., Azad C.S., Chaudhary P., Gambhir I.S. (2018). Role of Forkhead Box O (FOXO) transcription factor in aging and diseases. Gene.

[9] Brunet A., Bonni A., Zigmond M.J., Lin M.Z., Juo P., Hu L.S., Anderson M.J., Arden K.C., Blenis J., Greenberg M.E. (1999). Akt promotes cell survival by phosphorylating and inhibiting a Forkhead transcription factor. Cell.

[10] Zheng W.H., Kar S., Quirion R. (2000). Insulin-like growth factor-1-induced phosphorylation of the forkhead family transcription factor FKHRL1 is mediated by Akt kinase in PC12 cells. J. Biol. Chem..

[11] Greer E.L., Brunet A. (2005). FOXO transcription factors at the interface between longevity and tumor suppression. Oncogene.

[12] Biggs W.H., Meisenhelder J., Hunter T., Cavenee W.K., Arden K.C. (1999). Protein kinase B/Akt-mediated phosphorylation promotes nuclear exclusion of the winged helix transcription factor FKHR1. Proc. Natl. Acad. Sci. USA.

[13] Kops G.J., Dansen T.B., Polderman P.E., Saarloos I., Wirtz K.W., Coffer P.J., Huang T.T., Bos J.L., Medema R.H., Burgering B.M. (2002). Forkhead transcription factor FOXO3a protects quiescent cells from oxidative stress. Nature.

[14] Tran H., Brunet A., Grenier J.M., Datta S.R., Fornace A.J., DiStefano P.S., Chiang L.W., Greenberg M.E. (2002). DNA repair pathway stimulated by the forkhead transcription factor FOXO3a through the Gadd45 protein. Science.

[15] Low P. (2011). The role of ubiquitin-proteasome system in ageing. Gen. Comp. Endocrinol..

[16] Zhao J., Brault J.J., Schild A., Goldberg A.L. (2008). Coordinate activation of autophagy and the proteasome pathway by FoxO transcription factor. Autophagy.

[17] Rached M.T., Kode A., Xu L., Yoshikawa Y., Paik J.H., Depinho R.A., Kousteni S. (2010). FoxO1 is a positive regulator of bone formation by favoring protein synthesis and resistance to oxidative stress in osteoblasts. Cell Metab..

[18] Zhao J., Brault J.J., Schild A., Cao P., Sandri M., Schiaffino S., Lecker S.H., Goldberg A.L. (2007). FoxO3 coordinately activates protein degradation by the autophagic/lysosomal and proteasomal pathways in atrophying muscle cells. Cell Metab..

[19] Manolopoulos K.N., Klotz L.O., Korsten P., Bornstein S.R., Barthel A. (2010). Linking Alzheimer’s disease to insulin resistance: The FoxO response to oxidative stress. Mol. Psychiatry.

[20] Shen C., Cai G.Q., Peng J.P., Chen X.D. (2015). Autophagy protects chondrocytes from glucocorticoids-induced apoptosis via ROS/Akt/FOXO3 signaling. Osteoarthr. Cartil..

[21] Akasaki Y., Alvarez-Garcia O., Saito M., Carames B., Iwamoto Y., Lotz M.K. (2014). FoxO transcription factors support oxidative stress resistance in human chondrocytes. Arthr. Rheumatol..

[22] Akeson G., Malemud C.J. (2017). A Role for Soluble IL-6 Receptor in Osteoarthritis. J. Funct. Morphol. Kinesiol..

[23] Baar M.P., Brandt R.M., Putavet D.A., Klein J.D., Derks K.W., Bourgeois B.R., Stryeck S., Rijksen Y., van Willigenburg H., Feijtel D.A. (2017). Targeted Apoptosis of Senescent Cells Restores Tissue Homeostasis in Response to Chemotoxicity and Aging. Cell.

[24] Eelen G., Verlinden L., Maes C., Beullens I., Gysemans C., Paik J.H., DePinho R.A., Bouillon R., Carmeliet G., Verstuyf A. (2016). Forkhead box O transcription factors in chondrocytes regulate endochondral bone formation. J. Steroid Biochem. Mol. Biol..

[25] Li Y.S., Zhang F.J., Zeng C., Luo W., Xiao W.F., Gao S.G., Lei G.H. (2016). Autophagy in osteoarthritis. Jt. Bone Spine.

[26] Toh W.S., Brittberg M., Farr J., Foldager C.B., Gomoll A.H., Hui J.H., Richardson J.B., Roberts S., Spector M. (2016). Cellular senescence in aging and osteoarthritis. Acta Orthop..

[27] Giunta S., Castorina A., Marzagalli R., Szychlinska M.A., Pichler K., Mobasheri A., Musumeci G. (2015). Ameliorative effects of PACAP against cartilage degeneration. Morphological, immunohistochemical and biochemical evidence from in vivo and in vitro models of rat osteoarthritis. Int. J. Mol. Sci..

[28] Musumeci G., Szychlinska M.A., Mobasheri A. (2015). Age-related degeneration of articular cartilage in the pathogenesis of osteoarthritis: Molecular markers of senescent chondrocytes. Histol. Histopathol..

[29] Loeser R.F. (2009). Aging and osteoarthritis: The role of chondrocyte senescence and aging changes in the cartilage matrix. Osteoarthr. Cartil..

[30] Jeon O.H., Kim C., Laberge R.M., Demaria M., Rathod S., Vasserot A.P., Chung J.W., Kim D.H., Poon Y., David N. (2017). Local clearance of senescent cells attenuates the development of post-traumatic osteoarthritis and creates a pro-regenerative environment. Nat. Med..

[31] Kim J.H., Lee G., Won Y., Lee M., Kwak J.S., Chun C.H., Chun J.S. (2015). Matrix cross-linking-mediated mechanotransduction promotes posttraumatic osteoarthritis. Proc. Natl. Acad. Sci. USA.

[32] Van den Berg W.B. (2011). Osteoarthritis year 2010 in review: Pathomechanisms. Osteoarthr. Cartil..

[33] Takayama K., Kawakami Y., Kobayashi M., Greco N., Cummins J.H., Matsushita T., Kuroda R., Kurosaka M., Fu F.H., Huard J. (2014). Local intra-articular injection of rapamycin delays articular cartilage degeneration in a murine model of osteoarthritis. Arthritis Res. Ther..

[34] Carames B., Hasegawa A., Taniguchi N., Miyaki S., Blanco F.J., Lotz M. (2012). Autophagy activation by rapamycin reduces severity of experimental osteoarthritis. Ann. Rheum. Dis..

[35] Henrotin Y.E., Bruckner P., Pujol J.P. (2003). The role of reactive oxygen species in homeostasis and degradation of cartilage. Osteoarthr. Cartil..

[36] Henrotin Y., Kurz B., Aigner T. (2005). Oxygen and reactive oxygen species in cartilage degradation: Friends or foes?. Osteoarthr. Cartil..

[37] Lo Y.Y., Wong J.M., Cruz T.F. (1996). Reactive oxygen species mediate cytokine activation of c-Jun NH2-terminal kinases. J. Biol. Chem..

[38] Amin A.R., Attur M., Patel R.N., Thakker G.D., Marshall P.J., Rediske J., Stuchin S.A., Patel I.R., Abramson S.B. (1997). Superinduction of cyclooxygenase-2 activity in human osteoarthritis-affected cartilage. Influence of nitric oxide. J. Clin. Investig..

[39] Boileau C., Martel-Pelletier J., Moldovan F., Jouzeau J.Y., Netter P., Manning P.T., Pelletier J.P. (2002). The in situ up-regulation of chondrocyte interleukin-1-converting enzyme and interleukin-18 levels in experimental osteoarthritis is mediated by nitric oxide. Arthritis Rheum..

[40] Wu C., Zheng J., Yao X., Shan H., Li Y., Xu P., Guo X. (2014). Defective autophagy in chondrocytes with Kashin-Beck disease but higher than osteoarthritis. Osteoarthr. Cartil..

[41] Yu S.M., Kim S.J. (2014). Withaferin A-caused production of intracellular reactive oxygen species modulates apoptosis via PI3K/Akt and JNKinase in rabbit articular chondrocytes. J. Korean Med. Sci..

[42] Pelletier J.P., Fernandes J.C., Jovanovic D.V., Reboul P., Martel-Pelletier J. (2001). Chondrocyte death in experimental osteoarthritis is mediated by MEK 1/2 and p38 pathways: Role of cyclooxygenase-2 and inducible nitric oxide synthase. J. Rheum..

[43] Brandl A., Hartmann A., Bechmann V., Graf B., Nerlich M., Angele P. (2011). Oxidative stress induces senescence in chondrocytes. J. Orthop. Res..

[44] Wang X., Li F., Fan C., Wang C., Ruan H. (2011). Effects and relationship of ERK1 and ERK2 in interleukin-1beta-induced alterations in MMP3, MMP13, type II collagen and aggrecan expression in human chondrocytes. Int. J. Mol. Med..

[45] Sondergaard B.C., Schultz N., Madsen S.H., Bay-Jensen A.C., Kassem M., Karsdal M.A. (2010). MAPKs are essential upstream signaling pathways in proteolytic cartilage degradation--divergence in pathways leading to aggrecanase and MMP-mediated articular cartilage degradation. Osteoarthr. Cartil..

[46] Ashraf S., Cha B.H., Kim J.S., Ahn J., Han I., Park H., Lee S.H. (2016). Regulation of senescence associated signaling mechanisms in chondrocytes for cartilage tissue regeneration. Osteoarthr. Cartil..

[47] Shimada H., Sakakima H., Tsuchimochi K., Matsuda F., Komiya S., Goldring M.B., Ijiri K. (2011). Senescence of chondrocytes in aging articular cartilage: GADD45beta mediates p21 expression in association with C/EBPbeta in senescence-accelerated mice. Pathol. Res. Pract..

[48] Kim H.J., Park S.R., Park H.J., Choi B.H., Min B.H. (2005). Potential predictive markers for proliferative capacity of cultured human articular chondrocytes: PCNA and p21. Artif. Organs.

[49] Guarente L. (1999). Diverse and dynamic functions of the Sir silencing complex. Nat. Genet..

[50] Philipot D., Guerit D., Platano D., Chuchana P., Olivotto E., Espinoza F., Dorandeu A., Pers Y.M., Piette J., Borzi R.M. (2014). p16INK4a and its regulator miR-24 link senescence and chondrocyte terminal differentiation-associated matrix remodeling in osteoarthritis. Arthritis Res. Ther..

[51] Ashizawa S., Nishizawa H., Yamada M., Higashi H., Kondo T., Ozawa H., Kakita A., Hatakeyama M. (2001). Collective inhibition of pRB family proteins by phosphorylation in cells with p16INK4a loss or cyclin E overexpression. J. Biol. Chem..

[52] Lepetsos P., Papavassiliou A.G. (2016). ROS/oxidative stress signaling in osteoarthritis. Biochim. Biophys. Acta.

[53] Gao Y., Liu S., Huang J., Guo W., Chen J., Zhang L., Zhao B., Peng J., Wang A., Wang Y. (2014). The ECM-cell interaction of cartilage extracellular matrix on chondrocytes. BioMed Res. Int..

[54] Baker M.S., Feigan J., Lowther D.A. (1989). The mechanism of chondrocyte hydrogen peroxide damage. Depletion of intracellular ATP due to suppression of glycolysis caused by oxidation of glyceraldehyde-3-phosphate dehydrogenase. J. Rheum..

[55] Hauselmann H.J., Stefanovic-Racic M., Michel B.A., Evans C.H. (1998). Differences in nitric oxide production by superficial and deep human articular chondrocytes: Implications for proteoglycan turnover in inflammatory joint diseases. J. Immunol..

[56] Studer R.K., Levicoff E., Georgescu H., Miller L., Jaffurs D., Evans C.H. (2000). Nitric oxide inhibits chondrocyte response to IGF-I: Inhibition of IGF-IRbeta tyrosine phosphorylation. Am. J. Physiol. Cell Physiol..

[57] Yin W., Park J.I., Loeser R.F. (2009). Oxidative stress inhibits insulin-like growth factor-I induction of chondrocyte proteoglycan synthesis through differential regulation of phosphatidylinositol 3-Kinase-Akt and MEK-ERK MAPK signaling pathways. J. Biol. Chem..

[58] Yu S.M., Kim S.J. (2013). Production of reactive oxygen species by withaferin A causes loss of type collagen expression and COX-2 expression through the PI3K/Akt, p38, and JNK pathways in rabbit articular chondrocytes. Exp. Cell Res..

[59] Essers M.A., Weijzen S., de Vries-Smits A.M., Saarloos I., de Ruiter N.D., Bos J.L., Burgering B.M. (2004). FOXO transcription factor activation by oxidative stress mediated by the small GTPase Ral and JNK. EMBO J..

[60] Boccitto M., Kalb R.G. (2011). Regulation of Foxo-dependent transcription by post-translational modifications. Curr. Drug Targets.

[61] You H., Jang Y., You-Ten A.I., Okada H., Liepa J., Wakeham A., Zaugg K., Mak T.W. (2004). p53-dependent inhibition of FKHRL1 in response to DNA damage through protein kinase SGK1. Proc. Natl. Acad. Sci. USA.

[62] Kloet D.E., Burgering B.M. (2011). The PKB/FOXO switch in aging and cancer. Biochim. Biophys. Acta.

[63] Liu-Bryan R., Terkeltaub R. (2015). Emerging regulators of the inflammatory process in osteoarthritis. Nat. Rev. Rheum..

[64] Zhao X., Petursson F., Viollet B., Lotz M., Terkeltaub R., Liu-Bryan R. (2014). Peroxisome proliferator-activated receptor gamma coactivator 1alpha and FoxO3A mediate chondroprotection by AMP-activated protein kinase. Arthritis Rheum..

[65] Kim H.N., Han L., Iyer S., de Cabo R., Zhao H., O’Brien C.A., Manolagas S.C., Almeida M. (2015). Sirtuin1 Suppresses Osteoclastogenesis by Deacetylating FoxOs. Mol. Endocrinol..

[66] Takayama K., Ishida K., Matsushita T., Fujita N., Hayashi S., Sasaki K., Tei K., Kubo S., Matsumoto T., Fujioka H. (2009). SIRT1 regulation of apoptosis of human chondrocytes. Arthritis Rheum..

[67] Lei M., Wang J.G., Xiao D.M., Fan M., Wang D.P., Xiong J.Y., Chen Y., Ding Y., Liu S.L. (2012). Resveratrol inhibits interleukin 1beta-mediated inducible nitric oxide synthase expression in articular chondrocytes by activating SIRT1 and thereby suppressing nuclear factor-kappaB activity. Eur. J. Pharmacol..

[68] Kobayashi Y., Furukawa-Hibi Y., Chen C., Horio Y., Isobe K., Ikeda K., Motoyama N. (2005). SIRT1 is critical regulator of FOXO-mediated transcription in response to oxidative stress. Int. J. Mol. Med..

[69] Hoogeboom D., Essers M.A., Polderman P.E., Voets E., Smits L.M., Burgering B.M. (2008). Interaction of FOXO with beta-catenin inhibits beta-catenin/T cell factor activity. J. Biol. Chem..

[70] Almeida M., Han L., Martin-Millan M., O’Brien C.A., Manolagas S.C. (2007). Oxidative stress antagonizes Wnt signaling in osteoblast precursors by diverting beta-catenin from T cell factor- to forkhead box O-mediated transcription. J. Biol. Chem..

[71] Essers M.A.G., de Vries-Smits L.M.M., Barker N., Polderman P.E., Burgering B.M.T., Korswagen H.C. (2005). Functional interaction between beta-catenin and FOXO in oxidative stress signaling. Science.

[72] Wu Q., Huang J.H., Sampson E.R., Kim K.O., Zuscik M.J., O’Keefe R.J., Chen D., Rosier R.N. (2009). Smurf2 induces degradation of GSK-3beta and upregulates beta-catenin in chondrocytes: A potential mechanism for Smurf2-induced degeneration of articular cartilage. Exp. Cell Res..

[73] Litherland G.J., Hui W., Elias M.S., Wilkinson D.J., Watson S., Huesa C., Young D.A., Rowan A.D. (2014). Glycogen synthase kinase 3 inhibition stimulates human cartilage destruction and exacerbates murine osteoarthritis. Arthritis Rheum..

[74] Miclea R.L., Siebelt M., Finos L., Goeman J.J., Lowik C.W., Oostdijk W., Weinans H., Wit J.M., Robanus-Maandag E.C., Karperien M. (2011). Inhibition of Gsk3beta in cartilage induces osteoarthritic features through activation of the canonical Wnt signaling pathway. Osteoarthr. Cartil..

[75] Mattila J., Kallijarvi J., Puig O. (2008). RNAi screening for kinases and phosphatases identifies FoxO regulators. Proc. Natl. Acad. Sci. USA.

[76] Ruiz-Romero C., Calamia V., Mateos J., Carreira V., Martinez-Gomariz M., Fernandez M., Blanco F.J. (2009). Mitochondrial dysregulation of osteoarthritic human articular chondrocytes analyzed by proteomics: A decrease in mitochondrial superoxide dismutase points to a redox imbalance. Mol. Cell. Proteom..

[77] Carlo M.D., Loeser R.F. (2003). Increased oxidative stress with aging reduces chondrocyte survival: Correlation with intracellular glutathione levels. Arthritis Rheum..

[78] Regan E.A., Bowler R.P., Crapo J.D. (2008). Joint fluid antioxidants are decreased in osteoarthritic joints compared to joints with macroscopically intact cartilage and subacute injury. Osteoarthr. Cartil..

[79] Davies C.M., Guilak F., Weinberg J.B., Fermor B. (2008). Reactive nitrogen and oxygen species in interleukin-1-mediated DNA damage associated with osteoarthritis. Osteoarthr. Cartil..

[80] Storz P. (2011). Forkhead homeobox type O transcription factors in the responses to oxidative stress. Antioxid. Redox Signal..

[81] Matsuzaki T., Alvarez-Garcia O., Mokuda S., Nagira K., Olmer M., Gamini R., Miyata K., Akasaki Y., Su A.I., Asahara H. (2018). FoxO transcription factors modulate autophagy and proteoglycan 4 in cartilage homeostasis and osteoarthritis. Sci. Transl. Med..

[82] De Isla N., Charif N., Stoltz J.F. (2010). Are FoxO transcription factors implicated in osteoarthritis? Influence of Diacerhein. Bio-Med. Mat. Eng..

[83] Scott J.L., Gabrielides C., Davidson R.K., Swingler T.E., Clark I.M., Wallis G.A., Boot-Handford R.P., Kirkwood T.B., Taylor R.W., Young D.A. (2010). Superoxide dismutase downregulation in osteoarthritis progression and end-stage disease. Ann. Rheum. Dis..

[84] Middleton J.F., Tyler J.A. (1992). Upregulation of insulin-like growth factor I gene expression in the lesions of osteoarthritic human articular cartilage. Ann. Rheum. Dis..

[85] Dore S., Pelletier J.P., DiBattista J.A., Tardif G., Brazeau P., Martel-Pelletier J. (1994). Human osteoarthritic chondrocytes possess an increased number of insulin-like growth factor 1 binding sites but are unresponsive to its stimulation. Possible role of IGF-1-binding proteins. Arthritis Rheum..

[86] Verschure P.J., Marle J.V., Joosten L.A., Helsen M.M., Lafeber F.P., Berg W.B. (1996). Localization of insulin-like growth factor-1 receptor in human normal and osteoarthritic cartilage in relation to proteoglycan synthesis and content. Brit. J. Rheum..

[87] Watroba M., Maslinska D., Maslinski S. (2012). Current overview of functions of FoxO proteins, with special regards to cellular homeostasis, cell response to stress, as well as inflammation and aging. Adv. Med. Sci..

[88] Ludikhuize J., de Launay D., Groot D., Smeets T.J., Vinkenoog M., Sanders M.E., Tas S.W., Tak P.P., Reedquist K.A. (2007). Inhibition of forkhead box class O family member transcription factors in rheumatoid synovial tissue. Arthritis Rheum..

[89] Xu L., Sun C., Zhang S., Xu X., Zhai L., Wang Y., Wang S., Liu Z., Cheng H., Xiao M. (2015). Sam68 Promotes NF-kappaB Activation and Apoptosis Signaling in Articular Chondrocytes during Osteoarthritis. Inflamm. Res..

[90] Kayal R.A., Siqueira M., Alblowi J., McLean J., Krothapalli N., Faibish D., Einhorn T.A., Gerstenfeld L.C., Graves D.T. (2010). TNF-alpha mediates diabetes-enhanced chondrocyte apoptosis during fracture healing and stimulates chondrocyte apoptosis through FOXO1. J. Bone Miner. Res..

[91] Musumeci G., Mobasheri A., Trovato F.M., Szychlinska M.A., Graziano A.C., Lo Furno D., Avola R., Mangano S., Giuffrida R., Cardile V. (2014). Biosynthesis of collagen I, II, RUNX2 and lubricin at different time points of chondrogenic differentiation in a 3D in vitro model of human mesenchymal stem cells derived from adipose tissue. Acta Histochem..

[92] Ford-Hutchinson A.F., Ali Z., Lines S.E., Hallgrimsson B., Boyd S.K., Jirik F.R. (2007). Inactivation of Pten in osteo-chondroprogenitor cells leads to epiphyseal growth plate abnormalities and skeletal overgrowth. J. Bone Miner. Res..

[93] Hsieh S.C., Chen N.T., Lo S.H. (2009). Conditional loss of PTEN leads to skeletal abnormalities and lipoma formation. Mol. Carcinog..

[94] Morita K., Miyamoto T., Fujita N., Kubota Y., Ito K., Takubo K., Miyamoto K., Ninomiya K., Suzuki T., Iwasaki R. (2007). Reactive oxygen species induce chondrocyte hypertrophy in endochondral ossification. J. Exp. Med..

[95] Guidotti S., Minguzzi M., Platano D., Santi S., Trisolino G., Filardo G., Mariani E., Borzi R.M. (2017). Glycogen Synthase Kinase-3beta Inhibition Links Mitochondrial Dysfunction, Extracellular Matrix Remodelling and Terminal Differentiation in Chondrocytes. Sci. Rep..

[96] Rokutanda S., Fujita T., Kanatani N., Yoshida C.A., Komori H., Liu W., Mizuno A., Komori T. (2009). Akt regulates skeletal development through GSK3, mTOR, and FoxOs. Dev. Biol..

[97] Ye Z., Chen Y., Zhang R., Dai H., Zeng C., Zeng H., Feng H., Du G., Fang H., Cai D. (2014). c-Jun N-terminal kinase - c-Jun pathway transactivates Bim to promote osteoarthritis. Can. J. Physiol. Pharmacol..

[98] Sasaki H., Takayama K., Matsushita T., Ishida K., Kubo S., Matsumoto T., Fujita N., Oka S., Kurosaka M., Kuroda R. (2012). Autophagy modulates osteoarthritis-related gene expression in human chondrocytes. Arthritis Rheum..

[99] Shen C., Yan J., Erkocak O.F., Zheng X.F., Chen X.D. (2014). Nitric oxide inhibits autophagy via suppression of JNK in meniscal cells. Rheumatology.

[100] Ferdous A., Battiprolu P.K., Ni Y.G., Rothermel B.A., Hill J.A. (2010). FoxO, autophagy, and cardiac remodeling. J. Cardiovasc. Transl. Res..

[101] Salminen A., Kaarniranta K. (2009). SIRT1: Regulation of longevity via autophagy. Cell. Signal..

[102] Ng F., Tang B.L. (2013). Sirtuins’ modulation of autophagy. J. Cell. Physiol..

[103] Chang J., Wang W., Zhang H., Hu Y., Wang M., Yin Z. (2013). The dual role of autophagy in chondrocyte responses in the pathogenesis of articular cartilage degeneration in osteoarthritis. Int. J. Mol. Med..

[104] Lin Y.J., Seroude L., Benzer S. (1998). Extended life-span and stress resistance in the Drosophila mutant methuselah. Science.

[105] Orr W.C., Sohal R.S. (1994). Extension of life-span by overexpression of superoxide dismutase and catalase in Drosophila melanogaster. Science.

[106] Kampkotter A., Nkwonkam C.G., Zurawski R.F., Timpel C., Chovolou Y., Watjen W., Kahl R. (2007). Effects of the flavonoids kaempferol and fisetin on thermotolerance, oxidative stress and FoxO transcription factor DAF-16 in the model organism Caenorhabditis elegans. Arch. Toxicol..

[107] Holzenberger M., Dupont J., Ducos B., Leneuve P., Geloen A., Even P.C., Cervera P., Le Bouc Y. (2003). IGF-1 receptor regulates lifespan and resistance to oxidative stress in mice. Nature.

[108] Heinegård D. (2009). I-6 BASIC PERSPECTIVE ON THE ROLE OF BIOMARKERS IN THE DIAGNOSIS AND MONITORING OF OSTEOARTHRITIS. Osteoarthr. Cartil..

[109] Alvarez-Garcia O., Matsuzaki T., Olmer M., Masuda K., Lotz M.K. (2017). Age-related reduction in the expression of FOXO transcription factors and correlations with intervertebral disc degeneration. J. Orthop. Res..

[110] Ambrogini E., Almeida M., Martin-Millan M., Paik J.H., Depinho R.A., Han L., Goellner J., Weinstein R.S., Jilka R.L., O’Brien C.A. (2010). FoxO-mediated defense against oxidative stress in osteoblasts is indispensable for skeletal homeostasis in mice. Cell Metab..

[111] Prestwich T.C., Macdougald O.A. (2007). Wnt/beta-catenin signaling in adipogenesis and metabolism. Curr. Opin. Cell Biol..

[112] Iyer S., Ambrogini E., Bartell S.M., Han L., Roberson P.K., de Cabo R., Jilka R.L., Weinstein R.S., O’Brien C.A., Manolagas S.C. (2013). FOXOs attenuate bone formation by suppressing Wnt signaling. J. Clin. Investig..

[113] Iyer S., Han L., Bartell S.M., Kim H.N., Gubrij I., de Cabo R., O’Brien C.A., Manolagas S.C., Almeida M. (2014). Sirtuin1 (Sirt1) promotes cortical bone formation by preventing beta-catenin sequestration by FoxO transcription factors in osteoblast progenitors. J. Biol. Chem..

[114] Sakata S., Hayashi S., Fujishiro T., Kawakita K., Kanzaki N., Hashimoto S., Iwasa K., Chinzei N., Kihara S., Haneda M. (2015). Oxidative stress-induced apoptosis and matrix loss of chondrocytes is inhibited by eicosapentaenoic acid. J. Orthop. Res..

[115] Jallali N., Ridha H., Thrasivoulou C., Underwood C., Butler P.E., Cowen T. (2005). Vulnerability to ROS-induced cell death in ageing articular cartilage: The role of antioxidant enzyme activity. Osteoarthr. Cartil..

[116] Kishimoto H., Akagi M., Zushi S., Teramura T., Onodera Y., Sawamura T., Hamanishi C. (2010). Induction of hypertrophic chondrocyte-like phenotypes by oxidized LDL in cultured bovine articular chondrocytes through increase in oxidative stress. Osteoarthr. Cartil..

[117] Goodwin W., McCabe D., Sauter E., Reese E., Walter M., Buckwalter J.A., Martin J.A. (2010). Rotenone prevents impact-induced chondrocyte death. J. Orthop. Res..

[118] Mobasheri A., Matta C., Zakany R., Musumeci G. (2015). Chondrosenescence: Definition, hallmarks and potential role in the pathogenesis of osteoarthritis. Maturitas.

[119] Van der Kraan P., Matta C., Mobasheri A. (2017). Age-Related Alterations in Signaling Pathways in Articular Chondrocytes: Implications for the Pathogenesis and Progression of Osteoarthritis—A Mini-Review. Gerontology.

[120] Musumeci G., Castrogiovanni P., Trovato F.M., Imbesi R., Giunta S., Szychlinska M.A., Loreto C., Castorina S., Mobasheri A. (2015). Physical activity ameliorates cartilage degeneration in a rat model of aging: A study on lubricin expression. Scand. J. Med. Sci. Sports.

[121] Rahmati M., Nalesso G., Mobasheri A., Mozafari M. (2017). Aging and osteoarthritis: Central role of the extracellular matrix. Ageing Res. Rev..

[122] Zhou W., Chen L., Yang S., Li F., Li X. (2012). Behavioral stress-induced activation of FoxO3a in the cerebral cortex of mice. Biol. Psychiatry.

[123] Legendre F., Heuze A., Boukerrouche K., Leclercq S., Boumediene K., Galera P., Domagala F., Pujol J.P., Ficheux H. (2009). Rhein, the metabolite of diacerhein, reduces the proliferation of osteoarthritic chondrocytes and synoviocytes without inducing apoptosis. Scand. J. Rheumatol..

